# Young hospital pharmacists’ job stress and career prospects amidst the COVID-19 pandemic in China

**DOI:** 10.1186/s40545-021-00355-2

**Published:** 2021-08-06

**Authors:** Jiahao Wu, Jian Cai, Ming Fang, Yan Wang, Feng Xu

**Affiliations:** 1 Fengxian Hospital, Southern Medical University, Shanghai, China; 2 School of Pharmacy, Shanghai University of Medicine & Health Sciences, Shanghai, China; 3 Fengxian Mental Health Center, Shanghai, China

**Keywords:** COVID-19, Young pharmacists, Job stress, Intervention, China

## Abstract

**Background:**

Coronavirus disease (COVID-19) pandemic posed a critical threat to public health in the past year and has not been fully controlled so far. The nature of front-line young hospital pharmacists’ occupation puts them at an increased risk of contracting any contagious disease, including COVID-19. Recent survey indicated that hospital pharmacists in China are depressive, hostile amid the pandemic.

**Aim:**

The present investigation aims to understand the job stress among young hospital pharmacists during the outbreak of COVID-19 and to provide basic information for pharmacy managers to help young fellows to cope with job stress.

**Method:**

This study is adopting pharmacist job stress questionnaire as the key instrument of data collection through WJX App in mobile phone. Demographic information, career prospects and stress management proposals were obtained synchronously. Quantitative data were processed with SPSS. Significant differences were examined using analysis of variance and Chi-square analysis.

**Result:**

About 60% of 289 questionnaire respondents complained of job stress (178 respondents). According to the narrative description of the data, young pharmacists’ gender, education background, hospital grade, and specific work post had no significant effect on job stress difference. However, young pharmacists in different age-groups and professional titles showed different job stress. Pharmacists at the age of 31–35 complained more stress than the others. Pharmacists with high professional title (deputy chief pharmacist) complained more stress than the others. About 65% of 289 respondents had long-term plan for their practice, although 61% of young pharmacists felt troubled or worried with their future. As for stress management proposal, almost all young pharmacists hoped to improve their professional identity via raising their wages.

**Conclusion:**

More than half of young pharmacists suffer from job stress amidst the COVID-19 pandemic in China, and various intervention measures should be taken to relieve the stress and finally improve their social identity.

## Background

Currently, young hospital pharmacists face various heavy work demands, work–life conflicts, irregular work arrangements and heavy work pressure in China [1, 2]. To make matters worse, coronavirus disease (COVID-19) pandemic was a critical threat to public health in the past year or so and has not been fully controlled so far. Latest news cites that Chinese mainland reports 27 new COVID-19 cases, with 20 locally transmitted in Guangdong, and the results of detected gene sequencing in all infected patients in Guangzhou's latest outbreak are homologous, all from variants detected in India [3]. The nature of hospital pharmacists’ occupation puts them at an increased risk of getting any contagious COVID-19 [4]. Young pharmacists are usually on the front-line of the COVID-19 outbreak response and as such are more at risk of contracting this virus [5]. Besides, as social distancing and non-essential businesses are implemented to control the physical spread of COVID-19, these challenging measures increased stress, anxiety, depressive symptoms, and exacerbation of pre-existing mental illness [6]. Recent surveys reported negative psychological effects were induced by COVID-19 pandemic, which included stress, fear, anxiety, depression, burnout, hostility, and mental exhaustion in hospital pharmacists in China [7, 8].

Evidences showed that young pharmacists' job identity and self-satisfaction have been at a low level in China [9–11]. On the one hand, job burnout (such as repeated drug dispensing from day to day), lack of sense of accomplishment and low social position (compared with medical doctors in hospital) contributed to the low level [12]. On the other hand, the low income of young pharmacists has a great negative influence on their good life. Before year of 2017, there was still 15% drug price bonus in medical institution in China. At that time pharmacists were regarded as money-maker for hospital and they got more pay from hospital. Now, circumstances change with the passage of time. Drug price bonus in hospital is zero. The salary of hospital pharmacists has been greatly reduced. Low income brings about their survival challenge, and impairs their physical and mental health [13]*.* Last but not least, pharmacists especially young dispensing fellows face the challenge of professional transformation from simply dispensing to providing clinical pharmacy service. Many young dispensing pharmacists even have sprouted their intention to resign in the new context of COVID-19 pandemic. In short, as the health care system in China is different from that of other countries, the characteristics of pharmacist job stress might be different from that of other countries [14, 15]. Thus it is necessary to clarify the current situation of job stress among Chinese young hospital pharmacists during the outbreak of COVID-19. This work aims to provide basic information for pharmacy managers to help young fellows to cope with job stress.

## Methods

All data are divided into four sections and collected through WJX App in mobile phone. The first section was the demographic information of the respondents. The second section was job stress questionnaire. We adopted the validated pharmacist job stress questionnaire scale as the key instrument [16], which was composed of 12 job stress-related questions based on the various elements of the work (workload, work time, salary, relations with colleagues and leader, etc.) with five-level Likert scale (from strongly disagree, disagree, neutral, agree, to strongly agree, respectively, and grades assigning from 1 to 5 points, respectively). For each respondent the total score ranged from the minimum 12 to the maximum 60 points. The higher the sum value, the greater the job stresses. The third section dealt with personal viewpoints on profession development prospects. The fourth section was free comments on stress management proposals.

The survey was conducted from August 1 through September 30, 2020. Each mobile phone account is only allowed to submit once in order to avoid submitting questionnaires repeatedly. The study sample is young hospital pharmacists under the age of 35 years who work in the medical institutions at all levels in China. Participation was voluntary, and the responses were anonymous and no identifiable information was collected. Data differences were examined using analysis of variance and Chi-square test.

## Results

### Demographic data

A total of 289 survey respondents were effectively recovered with 219 females. 32 pharmacists were less than 25 years old and 153 pharmacists were over 31 years old. Among them, 213 pharmacists were assistant pharmacist or pharmacist, and only 5 pharmacists were deputy chief pharmacist. The other relevant demographic information and practice information of the respondents are given in Table 1.

**Table 1:** Demographic information and practice information of the respondents

Variable	Quantity
Gender	
Male	70 (24.22%)
Female	219 (75.78%)
Age	
Less than 25	32 (11.07%)
26–30	104 (35.99%)
31–35	153 (52.94%)
Education background	
Associate degree and below	40 (13.84%)
Bachelor degree	196 (67.82%)
Master degree or above	53 (18.34%)
Hospital grade	
Level 1	42 (14.53%)
Level 2	44 (15.22%)
Level 3	203 (70.24%)
Professional title	
Assistant pharmacist	40 (13.84%)
Pharmacist	173 (59.86%)
Pharmacist-in-charge	71 (24.57%)
Deputy chief pharmacist	5 (1.73%)
Chief pharmacist	0(0%)
Specific work post	
Dispensing pharmacy	190 (65.74%)
Clinical pharmacy service	55 (19.03%)
Pharmacy intravenous admixture services	28 (9.69%)
Other work posts	16 (5.54%)

### Job stress data

**Fig. 1:**
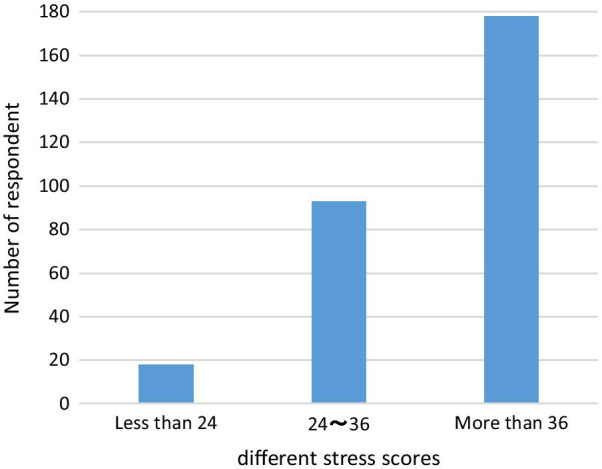
Varying degree job stress distribution of young pharmacists

Based on scale assignment statistics, score less than 24 points is regarded as no much job stress, score between 24 and 36 points is regarded as certain job stress, and score more than 36 points is regarded as higher job stress. In this survey, 18 respondents (6.23%) claimed they do not feel too much job stress (6.23%), 93 respondents (32.18%) felt a certain job stress, and 178 respondents (61.59%) expressed higher job stress (Fig. 1).

### Job stress *vs* gender, age, education background, hospital grade, professional title and specific work post

Our data (Table 2) showed that no gender, education background, hospital grade and specific work post differences in the job stress among young pharmacists in this survey. However, different age and different professional titles indicated a certain association with job stress. On one hand, young hospital pharmacist with different age-group showed significant differences in job stress levels (*P* < 0.01). The job stress score (36.34 ± 10.86) of pharmacists in 31- to 35-year-old age-group was higher than that of other age-group (28.28 ± 10.71 in less than 25-year-old age-group and 34.56 ± 9.23 in 26–30-year-old age-group), suggesting that job stress increased with length of employment. On the other hand, corresponding to length of employment, there was a significant difference in the job stress among young pharmacists with different professional titles. Their job stress scores were 29.88 ± 10.72, 35.07 ± 10.74, 36.59 ± 9.27, and 39.80 ± 8.35 for corresponding assistant pharmacist, pharmacist, pharmacist-in-charge, and deputy chief pharmacist, respectively (*P* < 0.01), suggesting deputy chief pharmacists confront more job stress for routine work, as well teaching and research.

**Table 2:** Job stress scores of the respondents

Variable	Job stress scores
Gender	
Male	36.46 ± 11.59
Female	34.28 ± 10.15
Age	
Less than 25	28.28 ± 10.71
26–30	34.56 ± 9.23
31–35	36.34 ± 10.86
Education background	
Associate degree and below	34.82 ± 9.54
Bachelor degree	34.42 ± 11.00
Master degree or above	36.57 ± 9.15
Hospital grade	
Level 1	34.12 ± 11.36
Level 2	32.43 ± 12.36
Level 3	35.46 ± 9.89
Professional title	
Assistant pharmacist	29.88 ± 10.72
Pharmacist	35.07 ± 10.74
Pharmacist-in-charge	36.59 ± 9.27
Deputy chief pharmacist	39.80 ± 8.35
Chief pharmacist	/
Specific work post	
Dispensing pharmacy	34.98 ± 10.58
Clinical pharmacy service	36.78 ± 9.47
Pharmacy intravenous admixture services	30.96 ± 11.58
Other work posts	32.63 ± 10.46

### Viewpoints of young pharmacists on their profession

Answers to three questions indicated the viewpoints of the young pharmacists on their profession prospect (Table 3). In general, among 289 respondents, more than 30% of the young pharmacists did not have long-term career plan for their work. And about 60% of young pharmacists were troubled or worried about their job future. More than half of young pharmacists will not choose pharmacist as profession a second time.

**Table 3:** Young pharmacists' options on pharmacist profession prospect issues

Questions	Options	Quantity
Do you still have a long-term career plan for pharmacists in the pandemic?	Yes	188 (65.05%)
	No	101 (34.95%)
How do you see your future?	Bright and confident	62 (21.45%)
	Feel troubled or worried	177 (61.25%)
	Have not considered	50 (17.3%)
If you had a chance again, would you still choose pharmacist as a career?	Yes	126 (43.6%)
	No	163 (56.4%)

Specifically, more than 70% of pharmacists in the 25–30 age-group were troubled or worried about their career future. All deputy chief pharmacists were troubled or worried about the career future.

More than half of young pharmacists in the 31–35 age-group would not choose pharmacist as occupation again. And more than 60% of deputy chief pharmacists would not choose the profession of pharmacists any more. All these data suggest that the profession of pharmacists was not a satisfying one for young people under the COVID-19 pandemic.

### Stress management proposal

Almost all young pharmacists hoped to improve their professional identity via raising their wages. Half of them hoped that pharmacy managers could organize team activity to release job stress. And about 50% of young fellows wanted to train or study further in order to master more skills to handle with professional transformation and to adapt the changing COVID-19 pandemic world.

## Discussion

COVID-19 pandemic significantly altered our routine, lifestyle, and stress level across the globe. This study explored the job stress of Chinese young hospital pharmacists during an outbreak of COVID-19 pandemic. The subjects of survey are young pharmacists who usually have been working for less than 10 years. We believe that if the young fellows are under great pressure during the unusual pandemic, it will have an adverse impact on their future career development.

In the past 5 years, a series of medical reform policies and rational drug use specifications have been issued intensively by the National Health Commission of China. First, the medical insurance payment mode convert (for example, disease diagnosis-related group, DRG) is advanced and currently being applied in medical institution national widely. Generic drugs have been advocated for use by all levels of government. Drug centralized procurement or drug quantity purchase is being executed all over the country [17]. Second, many new practice requirements are proposed to hospital pharmacist, such as controlling the medical expense rise via rational drug use. However, the pharmacist service fee has not yet been implemented despite the appeal for many years in China, meanwhile drug price zero bonus policy in medical institution has been implemented for couple of years. To a certain extent, these policies are absolutely negative bad news for young pharmacists. COVID-19 global pandemic officially declared on March 11, 2020 by the World Health Organization (WHO) furthermore brought about invisible pressure. Many front-line hospital pharmacists cited job stress and fear of the virus infection just like their foreign counterparts [18]. The COVID-19 pandemic is only the last straw to crush young pharmacists. Under this context, it is not a surprise that this survey showed that more than 60% young hospital pharmacist complained of high job stress and worried their job future during COVID-19 pandemic in China.

Specifically in this survey, we noted that the job stress increased with their age/professional title. The reasons are complex. In China, professional title in the hospital pharmacy has five categories, climbing from the bottom of assistant pharmacist through pharmacist, pharmacist-in-charge, deputy chief pharmacist, to the top of chief pharmacist. Pharmacists with different titles usually have different specific work post responsibility and requirement. At the present medical practice environment, there are more performance assessment indicators and more workload for deputy chief pharmacists, however, their income does not match their contribution correspondingly. Deputy chief pharmacists have to bear teaching and research work, as well as publishing papers to get promotion, which are tough pressure for many young pharmacists. A recent survey found that the COVID-19 pandemic has increased psychological stress among healthcare workers (including pharmacist) with 10 years or more work experiences in a central hospital in China [19], which is consistent to our results.

Although our data showed that no specific work post differences in the job stress among young pharmacists in this survey, 190 young dispensing pharmacists of all 289 young fellows poured out a lot of complaints besides the questionnaire investigation. They confessed that most outpatient pharmacies were equipped with automated dispensary systems, but automation does not effectively reduce the working time of dispensing pharmacists. In addition, since outpatient pharmacy is the final stop for hospital process, patients often turn their grievances and anger into complaints and finally vent their anger on the front-line dispensing pharmacist [20]. Young pharmacists have to bear the pressure of tense doctor–patient relationship [21]. In China, during the peak period of outpatient service, dispensing pharmacists are required to dispense in short time as soon as possible without any error. It is a great challenge. They fear being complained by patients [22]. They fear COVID-19 pandemic more and would prefer stay at home on leave without any income.

Therefore, it is of importance for pharmacy managers to help their young fellows in emotion management and release their job stress especially during the COVID-19 pandemic. As 90% of young pharmacists wish to raise salary, hospital pharmacy managers should advocate legislating for pharmacist service fee. Meanwhile hospital pharmacy managers should give their young fellows more opportunities to day-release course study. In general, the contribution of pharmacists in medical service should be recognized and physical and mental health care should be given simultaneously [23–25].

The study limitation is that job stress is measured using as a self-reported stress level.

## Conclusions

More than half of the young hospital pharmacists feel job stress. Job stress is inevitable. Pharmacy managers should help young fellows to alleviate their job stress and finally improve their professional social identity.
